# Molecular Mechanisms Underlying Pathological and Therapeutic Roles of Pericytes in Atherosclerosis

**DOI:** 10.3390/ijms231911663

**Published:** 2022-10-01

**Authors:** Siarhei A. Dabravolski, Alexander M. Markin, Elena R. Andreeva, Ilya I. Eremin, Alexander N. Orekhov, Alexandra A. Melnichenko

**Affiliations:** 1 Department of Biotechnology Engineering, ORT Braude College, Snunit 51, P.O. Box 78, Karmiel 2161002, Israel; 2 Petrovsky National Research Center of Surgery, Abrikosovsky Lane, 2, 119991 Moscow, Russia; 3 Laboratory of Cell Physiology, Institute of Biomedical Problems, Russian Academy of Sciences, Khoroshevskoye Shosse, 76a, 123007 Moscow, Russia; 4 Institute for Atherosclerosis Research, Osennyaya 4-1-207, 121609 Moscow, Russia; 5 Institute of General Pathology and Pathophysiology, 8 Baltiyskaya Street, 125315 Moscow, Russia

**Keywords:** pericyte, cardiovascular disease, atherosclerosis, diabetes, shear stress, angiogenesis

## Abstract

Pericytes are multipotent mesenchymal stromal cells playing an active role in angiogenesis, vessel stabilisation, maturation, remodelling, blood flow regulation and are able to trans-differentiate into other cells of the mesenchymal lineage. In this review, we summarised recent data demonstrating that pericytes play a key role in the pathogenesis and development of atherosclerosis (AS). Pericytes are involved in lipid accumulation, inflammation, growth, and vascularization of the atherosclerotic plaque. Decreased pericyte coverage, endothelial and pericyte dysfunction is associated with intraplaque angiogenesis and haemorrhage, calcification and cholesterol clefts deposition. At the same time, pericytes can be used as a novel therapeutic target to promote vessel maturity and stability, thus reducing plaque vulnerability. Finally, we discuss recent studies exploring effective AS treatments with pericyte-mediated anti-atherosclerotic, anti-inflammatory and anti-apoptotic effects.

## 1. Introduction

Pericytes, or perivascular cells, are branched cells located in the wall of capillary blood vessels, embedded within the microvascular basement membrane and wrapped around EC (endothelial cells). Pericytes can be found within most tissues of the body, in the vascular wall of both microvessels and large arteries and veins [1,2]. Pericytes support vascular network integrity and functionality through their close association with ECs. However, pericyte coverage and pericyte:EC ratio vary between different tissues, reaching 1:1 in the nervous system, where proper pericyte–EC communication is vital for blood–brain barrier function [3]. The pericyte:EC coverage in the heart is lower (about 1:2 to 1:3) [4], with only 1:100 in skeletal muscle [5], suggesting that the importance of pericytes for skeletal muscle functioning is less significant [6]. Pericytes are involved in a variety of physiological processes, such as differentiation, angiogenesis, immunomodulation, regeneration and blood flow regulation. These unique functional characteristics make pericytes a promising tool and target in regenerative medicine and treatment of many cardiovascular diseases [7].

Pericytes originate from HPSCs (human pluripotent stem cells), which develop into mesenchymal progenitor cells, and further differentiate into immature pericytes, MSCs (multipotent mesenchymal stem cells) and SMCs (smooth muscle cells). Immature pericytes can differentiate into type 1 and 2 pericytes [8]. Because of a common origin, pericytes share some MSC properties and can trans-differentiate into other cells of the mesenchymal lineage (such as osteocytes, chondrocytes, myocytes, adipocytes) and neural cells [9]. Identification and tracking of pericytes has been traditionally considered a difficult task. Currently, several molecular markers are used to identify pericytes, such as NG-2 (Neuron-glial antigen 2), CD13 (cluster of differentiation 13) and CD146, PDGFR-β (platelet-derived growth factor receptor), α-SMA (smooth muscle α-actin) and desmin. The presence of other markers, such as DLK-1 (delta-like homolog 1 or Endosialin) and RGS 5 (regulator of G protein signalling 5), would depend on the location, developmental stage or pathological state [10,11]. Further markers (such as CD31, CD34, Gli1, Sca1, STRO-1 and others) should be used to distinguish pericytes from other subpopulations of perivascular progenitor cells [12]. Identification of specific pericyte markers linking the tissue of origin with their regenerative potential for particular pathologies would ensure that the most effective pericytes are used for treatment.

The vital role of pericytes in vascular network integrity and functionality made them an important diagnostic and therapeutic target in a variety of cardiovascular pathologies, including AS (atherosclerosis) [13,14]. Currently, pericyte-based therapies are under intensive investigation in animal models of muscle damage, bone and skin injury, diabetes complications, chronic kidney injury and myocardial infarction [15,16]. In this review, we discuss pericytes’ linkage to the main risk factors of AS (such as shear stress, DM (diabetes mellitus) and hypercholesterolemia) and their role in atherosclerosis development and pathogenesis. Furthermore, we highlight several successfully used AS therapeutic strategies with pericyte-mediated effects.

## 2. Pericytes and Atherosclerosis Main Risk Factors

**Figure 1:**
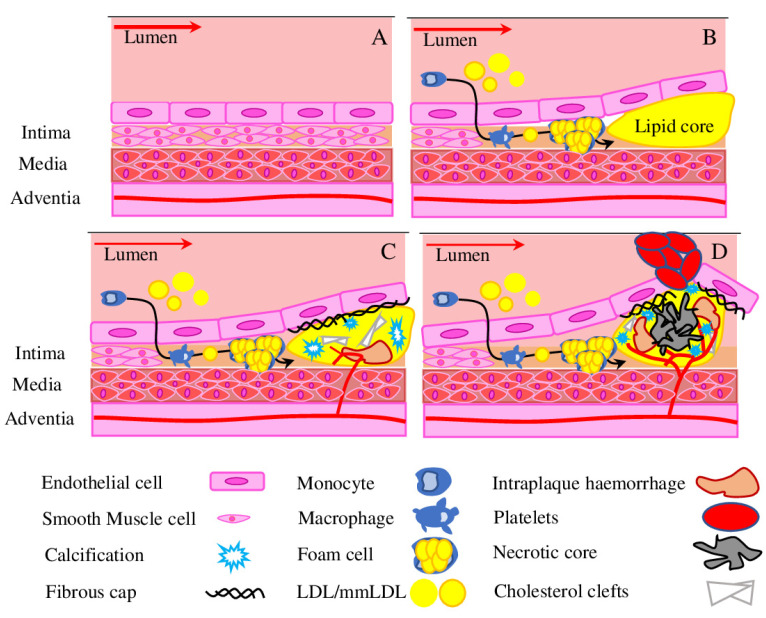
Atherosclerotic plaque formation. (**A**) Normal vessel. (**B**) Atherogenesis involves recruitment of inflammatory cells from blood, mostly represented by the monocyte. Upon activation, macrophages take up mmLDL, form foam cells and release pro-inflammatory factors. (**C**) A stable plaque is formed of a lipid core covered with a thin fibrous cap. Intraplaque haemorrhage, the extravasation of erythrocytes and inflammatory cells occurs because of immature leaky neovessels, often accompanied by deposition of cholesterol clefts and calcification. (**D**) Foam cells undergo apoptosis and necrosis, which results in a necrotic core formation. The thin fibrous cap of a vulnerable plaque is prone to rupture and can cause secondary complications such as thrombus formation. As disease progresses, the obstruction to the blood flow grows. Red arrow represents the intensity of the bloodstream.

Atherosclerosis is a multifactorial disease, described as a pathological remodelling of the arterial wall leading to its narrowing because of lipid accumulation and build-up of atheromatous plaque. Atherosclerosis progression is associated with various diseases, such as chronic kidney disease, peripheral artery disease, ischemic stroke and coronary artery disease. In total, different cardiovascular disease associated with advanced atherosclerosis account for 17.9 million deaths or 32% of all deaths per year worldwide, thus being the leading cause of mortality today [17]. Initial vascular remodelling manifests as diffuse thickening of the innermost vascular layer, which is driven by lipid accumulation (Figure 1), macrophage recruitment and uptake of mmLDL (multiple modified low-density lipoprotein), and their further transformation into the foam cells in the atherosclerotic plaque [18,19]. The increased number of macrophages and release of inflammatory cytokines, pro-apoptotic regulator BAX (Bcl-2-associated X protein) and TGF-β (transforming growth factor β) promote a shift of vascular smooth muscle cells into a more fibroproliferative condition, which further worsens the progression of the atherosclerotic lesion. In the later stages the thin fibrous cap is formed, it covers a necrotic lipid core and is often calcified. Such plaques are prone to rupture, which subsequently lead to a thrombus and vessel occlusion (Figure 1) [20].

Shear stress has been recognised as a crucial factor, contributing to the development of the initial lesion, thinning of the fibrous cap and vulnerable plaque rupture [21]. ECs are the major cell type able to sense and transduce mechano-signals through mechanosensors and mechanotransducers, localised in the membrane of ECs. The development of atherosclerotic plaques tends to occur at the site of arterial bifurcations and high curvatures, where haemodynamic forces (such as disturbed flow) acting through mechanosensors up-regulate pro-atherogenic and pro-inflammatory genes in ECs (YAP/TAZ pathway in ECs, VCAM1, HIF-1α, NF-κB, KLF2, KLF4 and others) [22]. KLF2 (Kruppel like Factor 2) is the best-characterised member of the KLF family and mechanosensitive transcription factors with well-studied roles in atherosclerosis [23]. Furthermore, recent experiments on atherosclerosis resistant BALB mice showed that disturbed blood flow is more prominent in promoting inflammation and atherosclerosis than a high-fat diet [24]. ADAMTS (disintegrin-like and metalloproteinase with thrombospondin motif) family proteases are known to regulate the structure and function of extracellular proteins in the extracellular matrix and blood [25], and are therefore connected to atherosclerosis progression. For example, a high level of ADAMTS7 was associated with increased plaque vulnerability in human carotid lesions [26]. Under shear stress, ECs show upregulated ADAMTS1, while pericytes demonstrated upregulated levels of TIMP3 (tissue inhibitor of matrix metalloproteinase) levels, an inhibitor of ADAMTS1. Also, ECs aligned in the direction of flow, while pericytes aligned perpendicularly, suggesting that they can sense direct flow. Therefore, pericytes are involved in vascular protection via expression of inhibitor of ADAMTS1 TIMP3, which prevents matrix degradation and maintains vascular stability [27].

Among multiple risk factors contributing to atherosclerosis development and progression, arterial hypertension, hypercholesterolemia and diabetes are intensively studied and considered to be major [28]. Arterial hypertension is the main driver of atherosclerosis development. However, depending on the localisation and experimental system used, hypertension has been associated with both an increase and a decrease in pericyte numbers [29]. Arterial hypertension is connected to vascular dysfunction via regulation of endothelial nitric oxide synthase, which produces the potent vasodilator NO. RAAS (renin–angiotensin–aldosterone system) is the key regulator of arterial blood pressure, fluid and electrolyte homeostasis, with Angiotensin II as the main effector peptide of this system [30]. Angiotensin II was shown to increase endothelial oxidative stress via NADPH oxidase–derived superoxide anion production and to induce endothelial dysfunction via CCR2/MCP-1 dependent macrophage recruitment to the vascular wall [31,32]. Hypercholesterolemia represents another risk factor in the development of cardiovascular disease. Increased cholesterol, LDL levels and LDL/HDL ratio are associated with a sedentary lifestyle and western diets, which lead to endothelial dysfunction and progression of the atherosclerotic lesion [33]. DM is another significant contributor to atherosclerosis development. The key DM feature—hyperglycaemia—is associated with increased ROS production, formation of AGEs (advanced glycation end products) and chronic inflammation [34].

### Pericytes in Diabetes

Pericytes are sensitive to metabolic abnormalities associated with DM (hyperinsulinemia, hyperglycaemia and dyslipidaemia), which causes pericyte dysfunction and contributes to the pathogenesis of many common complications of DM (such as retinopathy, peripheral artery disease, neuropathy and nephropathy) [34], including also atherosclerosis.

DM human hearts displayed capillary rarefaction and pericyte loss, associated with decreased contractility and increased stiffness. Also, in vitro experiments demonstrated that hyperglycaemia attenuated tube formation, migration, and pericyte attraction upon pro-angiogenic stimulation [35]. Similar results were obtained in T2DM mice, when skeletal muscle CD45- CD34- CD146+ pericytes were transplanted into ischemic hindlimb muscles to augment postischemic neovascularization. Pericyte transplantation augmented blood flow recovery and collateral artery enlargement in wild-type mice, but not in T2DM mice. Also, in this experiment, for the first time, the differentiation of pericytes into Schwann cells was detected in vivo [36]. Isolated mice cardiac NG2+ PDGFRb+ CD146+ CD34- CD31- CD45- pericytes demonstrated lipid droplet accumulation and decreased proliferation under high LDL levels. Similarly, elevated glucose levels triggered a pro-inflammatory response. Interestingly, high glucose treatment prevents cellular contraction, while high LDL had no such an effect [37].

Pericytes are also involved in the progression of DR (diabetic retinopathy) and DN (diabetic nephropathy)—the most common of DM complications [38]. DN is a chronic kidney disease characterised by renal hypertrophy, thickening of glomerular basement membranes, glomerular hypertension and glomerular hyperfiltration, which is manifested as albuminuria and hypertension [39]. Chronic hyperglycaemia promotes migration of peritubular pericytes away from the capillary into the interstitial space, thus destabilising capillaries and causing microvascular rarefaction and injury [40]. DR is a microvasculopathy associated with visual impairment and blindness, caused by microvascular damage to the inner lining of the retina. Pericytes are involved in maintenance of the blood–retinal barrier and environmental homeostasis in the retina. Losing intramural pericytes was associated with increased ER stress, vascular wall weakening, reduced vessel stability, generation of microaneurysms and subsequent retinal ischemia, causing severe vision loss [41,42]. Interestingly, aged ZSF1 rats have been characterised by obesity, hypertension and diabetes, but lack retina damage—the vascular density and pericyte coverage were not affected either by advanced age or DM [43]. On the other hand, a single intravitreal injection of CD140B, CD146, NG2 pericytes into DM Brown Norway rats was shown to functionally integrate into the damaged host retinal blood vessels and stabilise the blood-retinal barrier breakdown [44].

As discussed in this section, the formation of atherosclerotic lesions can be stimulated by multiple interdependent risk factors (such as arterial hypertension, shear stress, diabetes mellitus, hypercholesterolemia), which cause pericyte dysfunction, endothelial damage and decrease vascular stability.

## 3. Advanced Atherosclerosis

Advanced atherosclerotic lesions are characterised by the presence of necrotic cores with thin fibrous caps, deposition of cholesterol clefts, IPH (intraplaque haemorrhage), inflammatory cells and calcifications (Figure 1C,D) [45]. Inflammation is the key factor in every stage of atherosclerosis development and progression [46,47]. It is known, that macrophages and pericytes engulf mmLDL and turn into lipid filled foam cells, activate cytokine production and promote the influx of other inflammatory cells and their retention in the plaque [48]. Recent research has provided new details helping to understand the formation of unstable lesions. For example, it was shown that plaque angiogenesis and IPH play a central role in the formation of unstable lesions [49]. The role of smooth muscle cells in atherosclerotic plaque development and progression was recently reconsidered, suggesting their wider targeting for therapeutic intervention [50]. Similarly, the role of calcification for plaque stability was clarified, thus micro-calcifications lead to plaque destabilisation, while extended calcification can stabilise plaques [51].

Further, we focus on the advanced stages of atherosclerosis and the involvement of pericytes in plaque neovascularization, inflammation, and effects of calcification and IPH on plaque progression and instability.

### 3.1. Hypoxia Drives Angiogenesis

Hypoxia is the main driver of angiogenesis in atherosclerosis. Hypoxia promotes monocyte/macrophage survival and mmLDL uptake by macrophages, up-regulates the main regulators (VEGF (vascular endothelial growth factor), eNOS (endothelia nitric oxide synthase), and PDGF (platelet-derived growth factor), HIF-1 (hypoxia-inducible factor-1)) and increases ROS production [52]. Furthermore, the hypoxic state activates pro-apoptotic pathways and inflammatory responses, which result in cell death and expansion of the necrotic core, recruitment of inflammatory cells and expansion of the foam cell population (Figure 1D) [53]. The NF-κ B (nuclear factor-kB) is another crucial player in HIF-1/VEGF crosstalk. The activation of NF-κB can increase the release of IL-6 and, subsequently, activate STAT-3, leading to VEGF secretion, which could affect NF-kB expression via an auto-feedback loop. Also, the p50 and p65 NF-κ B subunits can bind to the HIF-1α promoter in response to hypoxia and modulate its expression [54]. The complete mechanism of molecular interaction between HIF-1, NF-κ B and VEGF under hypoxic conditions is not yet fully understood, but known features highlight the complex and interrelated connection between hypoxia and inflammatory signalling cascades in atherosclerosis. The connection between hypoxia and neovascularization is best studied in cancer, where processes of cancerisation and endothelialisation are crucial for cancer progression and metastasis [55].

### 3.2. Angiogenesis in the Atherosclerotic Plaque

**Figure 2:**
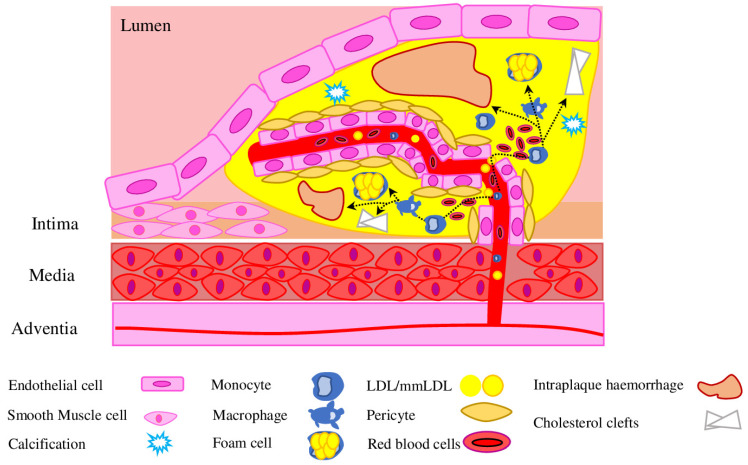
Pericytes in plaque angiogenesis. Plaque neovessels grow predominantly from vasa vasorum under the influence of hypoxia and a VEGF gradient. To become a mature functional vessel, the vessel has to be covered by pericytes. Poor pericyte coverage causes formation of immature leaky neovessels, which allow circulating cells to enter surrounding areas and form an intraplaque haemorrhage, enhance inflammation, calcification and cholesterol deposition.

Pathological angiogenesis of the vessel wall is a consistent feature of atherosclerotic plaque development and progression of the disease. The current view suggested that most of the intraplaque neovessels originate from the adventitial side, with little contribution from the luminal side. Early intimal damage induces neovascularization of vasa vasorum within the adventitia. However, despite initial expansion and penetration into the media and plaque of advanced lesions, neovessels lack proper coverage by pericytes, which leads to hyperpermeability (Figure 2) [56]. Pericytes are crucial for proper neovessel development and maturation [57], however, under AS-associated physiological conditions, PC respond to pro-angiogenic signals (VEGF, Ang2) and detach from the basement membrane, which leads to disruption of EC cellular junctions (VE-cadherins and claudins) [58]. Therefore, intraplaque neovascularization represents a new source for more mmLDL, pro-inflammatory cytokines and pro-angiogenic signals, which leads to further sprouting in the plaque, formation of a network of growing microvessels and plaque destabilisation. In the later stages, the thin cap fibroatheroma is formed, which comprises a large, lipid-rich necrotic core and a highly inflamed mass with intraplaque neovessels surrounded by a thin fibrous cap [59]. Most morbidity and mortality cases (such as myocardial infarction, stroke and sudden death) are associated with plaque rupture or erosion with subsequent luminal thrombosis [60].

### 3.3. Intraplaque Haemorrhage

Neovessels in vulnerable plaques are immature, characterised by a discontinuous basement membrane, a low number of tight junctions between the ECs and relatively poor pericyte coverage, thus, are highly susceptible to the leakage of circulating cells and lead to IPH (intraplaque haemorrhage) (Figure 2) [61]. Therefore, presence and stage of neovascularisation/haemorrhage serve as a good indicator of lesion instability and a higher risk of rupture [62]. VEGF and its main receptors VEGFR-1 and VEGFR-2 are key players responsible for EC proliferation and tube formation, pericyte attachment and detachment during neovessel initiation and growth [63]. The Ang/Tie signalling pathway plays an important role in the neovessel maturation process. Tie 2 (Tyrosine-Protein Kinase Receptor TIE-2) receptor is expressed on both pericytes and ECs, Ang-1 (Angiopoietin 1) is expressed on pericytes, while Ang-2 is expressed only by ECs. Ang-2 destabilises the interactions between pericytes and ECs, thus allowing vessels to grow. Ang-1 and PDGF stabilise the junctions between the ECs and pericytes, promoting vessel maturity and stability and reducing leakiness [64]. The balance between Ang-1 and Ang-2 expression is crucial for plaques microvascular density, while PDGF-BB and its receptor (PDGFR)-β, responsible for pericyte recruitment and stabilisation, are important for neovessels’ permeability, fragility, and impaired perfusion [65].

Red blood cells (RBC) are rich in unesterified cholesterol, haemoglobin and cell membrane components. Upon quick lysis in oxidative intraplaque environment, red blood cells participate in cholesterol accumulation and attract monocytes and neutrophils to the plaque [66]. In particular, the level of glycosylated haemoglobin (HbA1c) correlates with the fibrous cup thickness, thus contributing to the plaque instability [67]. Cholesterol of RBC membranes and free cholesterol are retained within the plaques and form cholesterol crystals, which are associated with intraplaque neovascularization and increased plaque susceptibility to haemorrhage and rupture (Figure 2) [68].

### 3.4. Pericytes in Vascular Calcification

Vascular calcification (VC) is an important complication of atherosclerosis, significantly contributing to cardiovascular morbidity and mortality worldwide. VC is characterised as a pathological accumulation of calcium phosphate crystals in the vessel wall or inside of atherosclerosis plaque. As an active and well-orchestrated process, VC shares numerous pathways with normal physiologic bone formation, suggesting a crosstalk between bones and vascular systems [69]. This process termed as “bone-vascular axis” is mediated through a variety of osteoblast-like cells, which could be derived from other cells: progenitor cells, ECs in the aortic intima, VSMCs in the media, pericytes in the microvessels, myofibroblasts in the adventitia and calcifying vascular cells (CVCs) [reviewed in [70]]. Inflammation, high blood glucose levels, lipid metabolism disorders, insulin abnormalities, expression of diverse growth factors, bone-related and matrix proteins initiate and regulate mineralisation process [reviewed in [71]].

Examination of atheromatous plaques showed asymptomatic plaques were associated with a higher presence of OM (osteoid metaplasia, or bone-like vascular calcification), levels of OPG (osteoprotegerin) and pericytes. Similarly, the levels of circulating OPG were higher in the plasma of asymptomatic patients. Further experiments in vitro showed that pericytes secreted OPG and underwent osteoblastic differentiation [72]. Later, these results were confirmed, when it was demonstrated that pericytes surrounding OM under inflammatory stress (IL-6 and TNF-α) were prone to secrete OPG and stimulate mineralisation. However, in the absence of inflammation, pericyte supernatant inhibited the osteogenic differentiation of human mesenchymal stem cells and of SMC [73]. While OPG is strongly expressed in stable, calcified plaques [74], the close association between inflammation and pericyte’s secreting OPG represents an interesting potential therapeutic target to prevent and treat vascular calcification. Similarly, pericytes and pericyte-like cells isolated from atherosclerotic ApoE^−/−^ mice showed significantly more in vitro osteogenesis and chondrogenesis in comparison to control cells. In particular, IL-6 treatment resulted in a significant increase in glycosaminoglycan deposition and expression of characteristic chondrogenic genes, suggesting a significant contribution of atherosclerotic inflammatory milieu to calcification [75].

## 4. Role of Pericytes in Plaque Stabilisation

Vulnerable atherosclerotic plaques are characterised by a thin, inflamed fibrous cap over a large lipid core, containing an increased number of inflammatory cells and frequently with intraplaque haemorrhaging. Vulnerable atherosclerosis plaques are prone to rupture and are a major cause of ACS (acute coronary syndrome) [76]. Intraplaque angiogenesis is a strong indicator of rapid plaque progression and risk of rupture and thrombosis [77]. While VEGF family proteins, FGF (fibroblast growth factor) family, and PDGF family are the main regulators of angiogenesis, the MMPs family (matrix metalloproteinases) and their inhibitors TIMP (tissue inhibitors of matrix metalloproteinase) serve as a diagnostic marker to access plaque development stage and stability [78].

Recent research on a rabbit model of atherosclerotic plaques has investigated a new strategy to improve plaque stability with local delivery of VEGF-A, FGF-2 and PDGF-BB by lentivirus into the atherosclerotic plaque. It is known, that leaky plaque neovessels and intraplaque haemorrhage co-localize with VEGF/VEGFR2 and angiopoietins. As expected, site-specific overexpression of VEGF-A/FGF-2 increased the number of immature neovessels and subsequently caused intraplaque haemorrhage and inflammatory cell infiltration, thus increasing plaque vulnerability. On the contrary, FGF-2/PDGF-BB overexpression increased neovessel pericyte coverage, which resulted in the development of mature and functional neovessels. Also, FGF-2 and PDGF-BB enhance VEGFR2 degradation by increasing epsin-2 expression [79]. Epsins are known as a family of endocytic clathrin adaptors involved in regulating endothelial cell VEGF signalling, especially investigated for their role in tumour development and progression [80]. Interestingly, FGF-2 alone significantly increased epsin-1/2 expression in pericytes, whereas FGF-2/PDGF-BB treatment increased only epsin-2 expression. These results demonstrated that FGF-2/PDGF-BB treatment promoted the function and maturation of plaque neovessels via improved pericytes function, suggesting a novel potential treatment strategy to improve plaque stability [79].

Similarly, experiments on hypercholesterolaemic ApoE3*Leiden mice demonstrated that VEGF treatment resulted in increased plaque angiogenesis. On the other hand, the blockade of VEGFR2 with monoclonal blocking antibodies showed a 44% decrease in intraplaque haemorrhage and reduced extravasated erythrocytes and macrophage content (80% and 30%, respectively), and increased collagen and SMCs content (54% and 123%, respectively) compared to controls. Also, VEGFR2 blocking-mediated plaque stabilisation was accompanied by decreased VEGF and Ang-2 expression, while the expression of Connexin 40 and pericyte coverage of the capillary sprouts were increased [81]. Interestingly, other research showed that the related Connexin 43 is crucial for pericyte-EC communication during early vessel formation [82]. While different connexins may compensate each other, the correction of pericytes coverage via connexins level modulation may be a promising revascularisation/vascularisation strategy.

Furthermore, these data are in accordance with early works, proving presence of a continuous three-dimensional network formed by pericyte-like cells in subendothelial intima [83,84]. Cells in such networks actively communicate through the gap junctions and form multicellular structures in a form of clusters [85]. However, multiply modified LDL (oxidised, desialylated and other modifications) associated with atherosclerosis lesions drastically reduced the intercellular communication by 1.9–8.8-fold. Accordingly, the level of connexin 43 was decreased three-fold in the initially damaged intima and was 17- and 25-fold lower in fibrolipid plaques and fibrotic plaques, respectively [86]. Therefore, these data explain the role of accumulated modified lipids in the reduction in gap junctions and disruption of intercellular network communication, subsequently leading to disease progression.

PDGF-BB recruits and guides pericytes towards the ECs of the developing vessel. The short PDGF-BB isoform obtained by deletion of the retention motif caused pericyte loss and subsequently led to increased microvascular leakage of the blood–brain barrier [87]. The whole-body PDGF-BB knockout is embryonic lethal because of widespread bleeding. Similarly, the application of PDGF receptor tyrosine kinase inhibitors is accompanied by excessive bleeding [88]. Naturally, PDGF-BB is produced and secreted by various cells (including vascular ECs and macrophages) with and without the C-terminal retention motif, which defines it as a cell-associated or soluble isoform, respectively. However, both isoforms are biologically active and stimulate fibroblast and SMC proliferation and ECM (extracellular matrix) formation [89]. Deletion of the PDGF-BB retention motif in murine atherosclerosis models resulted in increased plaque size and collagen content, reduced plaque macrophage content and MMP activity, while microvessel density and intraplaque haemorrhage were unaffected. Interestingly, plaque inflammation was decreased in Pdgfb^ret/ret^ mice, while systemic leukocytosis was observed only in Pdgfb^ret/ret^ mice on a high cholesterol diet. Also, overall reduced body weight gain, associated with lower fat deposition in liver and adipose tissue, was observed in mutant mice. Therefore, instead of affecting plaque or adventitial microvessel number and leakage, the deletion of PDGF-BB retention motifs demonstrated protective vascular and metabolic effects, accompanied by reduced plaque inflammation [90].

Different results were obtained in the rabbit atherosclerosis model under high cholesterol conditions, where the expression of VEGF-A, VEGFR-2, FGF-2, FGFR-1 (FGF receptor 1), PDGF-BB, TNF-α (tumour necrosis factor alpha) and the microvessel density were significantly elevated, while the level of PDGFR-β showed no significant change of expression. Also, plaque neovessels were leaky because of the decreased pericyte coverage. Interestingly, the levels of VEGF-A/VEGFR-2 and FGF-2/FGFR-1 correlate with microvessel density, macrophage content and TNF-α level. Therefore, demonstrating that high expression of FGF-2/FGFR-1 and VEGF-A/VEGFR-2 but not of PDGF-BB/PDGFR-β may contribute to intraplaque inflammation, immature angiogenesis and plaque instability [91].

**Figure 3:**
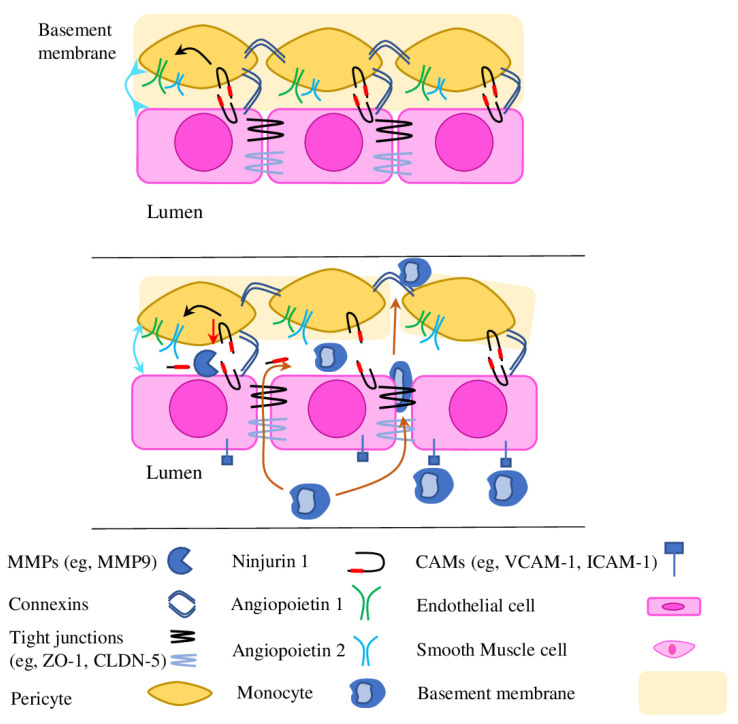
The role of adhesion molecules in pericyte–pericyte and pericyte–ECs communication. Disruption of the PC/PC and PC/EC signalling causes a wide variety of changes: (1) pericyte loss; (2) EC activation and extravasation of immune cells (orange arrows); (3) MMP activation leads to loss of tight and gap junctional proteins (occludens, claudins and connexins) and Ninj1 cleavage, changes in cells’ organisation and widening EC/EC junctional gaps, disrupting PC/PC connection and PC/EC association (cyan arrows); (4) MMP9 degrades basement membrane, which facilitates further PC loss and EC desquamation, morphologic changes to ECs with cellular hyperplasia and aberrant angiogenesis. The level of Ninj1 expression regulates Ang proteins expression (black arrows) and, subsequently, EC/PC association.

Ninjurin 1 (nerve injury-induced protein 1, or Ninj1) is a cell adhesion molecule responsible for cell–to–cell interactions between different cells and involved in many molecular pathways and processes (neuroinflammation, angiogenesis, muscle and osteoclasts development and others) [92,93]. Ninj1 is also involved in ECs\pericytes interaction and serves as a negative regulator of neovessel formation. Thereby, pericyte-specific Ninj1 down-regulation enhanced pericytes-mediated angiogenic effects and enhanced VEGF and Ang-1 production, while Ninj1 overexpression opposes those effects [94]. Later research has found an enhanced expression of Ninj1 in ischemic tissues after mouse hindlimb ischemia. Contrasting with previous research, Ninj1 overexpression enhanced expression of Ang-1 and stimulated the association of pericytes with ECs and subsequent formation of a capillary-like structure. Respectively, pericyte-specific Ninj1 knockout enhances Ang-2 expression (Ang-1 antagonist) and reduces the formation of blood-circulating functional vessels within ischemic tissues (Figure 3) [95].

Recently, the pericyte-specific Ninj1 deletion mouse was generated to show the central role of Ninj1 in adventitial angiogenesis and vascular remodelling of injured vessels. Mutant mice were characterised by an increased number of infiltrated macrophages in adventitia and expression of pro-inflammatory cytokines, vascular leakiness and enhanced intimal hyperplasia [96]. Interestingly, recent research on Apoe^−/−^ mice lacking systemic Ninj1 expression (Ninj1^−/−^Apoe^−/−^) demonstrated that soluble Ninj1 is an atheroprotective protein. Ninj1 expression correlates with atherosclerotic lesions, and the Ninj1-deficient macrophages promoted pro-inflammatory gene expression, the same as bone marrow-specific and whole-body Ninj1 deficiencies increased monocyte recruitment and macrophage accumulation in atherosclerotic lesions. Ninj1 is known as a direct cleavage substrate for MMP9, which generates a soluble form of Ninj1. Treatment with soluble Ninj1 mimicking peptides Ninj _11-56_ and Ninj1_26-37_ reduced macrophage inflammation and monocyte recruitment in atherosclerosis in both in vitro and in vivo conditions, thus confirming its atheroprotective properties [97].

Advanced stages of atherogenesis associated with angiogenesis in atherosclerotic plaques, calcification, cholesterol clefts and IPH, which strongly correlated with atherosclerotic plaque progression, instability and rupture. Discussed results indicate the importance of the crosstalk between capillary cells and pericytes and the role of pericyte coverage in survival and repair of ECs, leaky plaque neovessels stabilisation. Also, presented results provide a new scientific framework for the development of anti-angiogenic pericyte-based strategies to prevent atherosclerotic plaque progression and instability.

## 5. AS Treatments with Pericyte-Mediated Effects

The treatment of AS was addressed in countless studies; despite the constant development of new therapeutic strategies and drugs (such as nanomedicine, decoy and anti-platelet drug-loaded targeted technologies) [98,99,100], lipid-lowering and anti-inflammatory drugs are currently used as the principal drug in the first- and second-line treatments to prevent coronary artery disease [101,102]. However, pericytes may suggest some new diagnoses and therapeutic strategies for AS and other vascular pathologies [13]. Interestingly, both long-time known and new drugs have at least part of their beneficial effects provided through the pericytes.

A popular lipid-lowering drug atorvastatin was demonstrated to effectively reduce cholesterol levels, vessel remodelling and inflammation in ApoE3*Leiden mice on high cholesterol dies. Also, the presence of immature vessels was decreased by 34% and IPA by 30%. The molecular mechanism was defined in in vitro experiments, where culture of mice aortic-segments treated with atorvastatin demonstrated inhibited Ang2 release and higher expression of VE-Cadherin, accompanied by decreased VE-Cadherin (Y685) phosphorylation in ECs, which resulted in increased pericyte coverage [103]. VE-Cadherin is crucial for proper vascular development, its close association with pericyte coverage and angiogenesis was demonstrated also in tumours [104] and retinal vasculature development [105].

**Figure 4:**
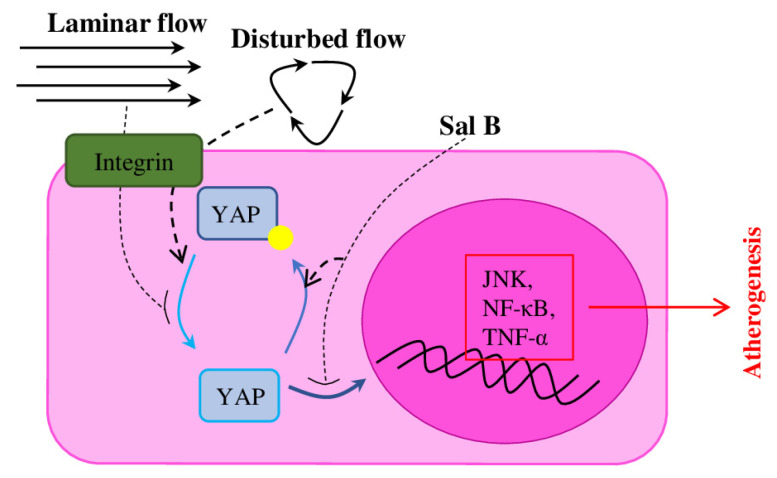
Haemodynamics regulates YAP/TAZ signalling in ECs. YAP/TAZ is a haemodynamics-induced mechanotransduction system. Laminar flow suppresses transactivation activity of YAP/TAZ, while disturbed flow reduces pYAP. Suppression of YAP activity with Sal B results in decreased expression of YAP/TAZ target genes (including pro-inflammatory cytokines) and retards atherosclerosis.

Recent research showed that treatment with Sal B (salvianolic acid B), a phenolic acid compound extracted from *Salvia miltiorrhiza*, effectively attenuates atherosclerosis via suppression of the YAP/TAZ/JNK signalling pathway in ECs and pericytes [106]. YAP (Yes-associated protein)/TAZ (transcriptional coactivator with PDZ-binding motif), effectors of the Hippo pathway, known for their involvement in organ size control, tissue homeostasis, cell proliferation and tumour suppression, recently also have been identified as mediators for mechanical stimuli [22]. Endothelial YAP/TAZ activity is regulated by blood flow, and YAP/TAZ inhibition suppresses inflammation and delay atherogenesis (Figure 4) [107]. Sal B suppresses the expression of ROS and pro-inflammatory cytokines (IL-1β, IL-6 and TNF-α) [108], and provides neuroprotective activities, which makes it a promising treatment for dementia and Alzheimer’s diseases [109]. Therefore, Sal B treatment in in vitro cell culture protects ECs and pericytes from oxidative stress and apoptosis, decreases the expression of YAP/TAZ pathway proteins (JNK, NF-κ B and TNF-α). Application of Sal B on the ApoE^−/−^ atherosclerosis mouse model reduced the lesion size and decreased the oxLDL levels in serum and the expression of pro-inflammatory cytokines (TNF-α, IL-1β and IL-6) [106].

Similarly to Sal-B, application of ghrelin improves endothelial dysfunction and also reduces vascular leakage in atherosclerotic mice by regulating inflammatory and apoptotic p38 MAPK-JNK signalling pathways in pericytes [110]. Ghrelin is a 28 amino acid peptide hormone, which alleviates mitochondrial dysfunction and endothelial apoptosis through the inhibition of JNK1/2 and p38 signalling pathway [111]. Also, ghrelin demonstrated atheroprotective and vascular function beneficial effects on HFD fed mice model, where ghrelin overexpression prevented vascular dysfunction, reduced vascular oxidative stress, vessels lipid accumulation and plaque formation [112]. Ghrelin administration to ApoE^−/−^ mice on HFD reduced serum oxLDL level, decreased the levels of pro-inflammatory cytokines (TNF-α, MCP-1 and IL-6), suppressed p38 MAPK-JNK signalling pathway in brain tissue, which resulted in reduced vascular leakage. Investigation of the ghrelin effects on the culture of isolated brain microvascular oxLDL-stimulated pericytes showed similar effects on p38 MAPK-JNK pathways and cytokines levels. Additionally, identified reduction in the Bax/Cleaved Caspase-3 and upregulation of Bcl-2 expression suggested a crucial role of ghrelin in the inhibition of inflammation and apoptosis of pericytes [110].

Application of multi-target traditional herbal medicines, such as SMYA (Si-Miao-Yong-An), which is composed of *Scrophularia ningpoensis*, *Lonicera japonica*, *Angelica Sinensis* and *Glycyrrhiza uralensis*, was an effective AS treatment. In particular, SMYA provided anti-inflammatory and anti-oxidative effects, inhibits the angiogenesis in plaques and decreases the AS plaque area in ApoE^−/−^ mice. Furthermore, SMYA-mediated regulation of HIF-1α and Ang1/Tie signalling pathways, which stabilised AS vulnerable plaques, promoted VV (vasa vasorum) maturation and suppressed VV neovascularization [113]. Interestingly, SMYA provided more pronounced effects compared to simvastatin treatment, it significantly reduced plaque area and the intima medium thickness. VV maturation effect was achieved through increased pericytes recruitment. On the molecular level SMYA was shown to increase the expression of Dll4 (Delta Like Canonical Notch Ligand 4), Notch1 (Notch homolog 1) and Hey1, while the expression of VEGF was decreased [114]. Other research has demonstrated that Hey1 is a crucial regulator of Notch signalling [115], and interplay between Dll4/Notch1 and VEGF signalling pathways are essential for regulation of vessels angiogenesis/regression [116,117]. In total, cooperation of YAP, Dll4/Notch and VEGF signalling pathways creates a robust and effective system maintaining vascular structure and function [118].

The solid understanding of molecular mechanisms involved in the atherosclerosis lesion development and its progression towards advanced stages is necessary to fully realise therapeutic potential hidden in known medications and natural bioactive compounds. Only during the last decade, pericytes have been recognised as an important therapeutic target, which could greatly affect the course of atherosclerosis to a more favourable one. However, to fully assess the effectiveness of the pericyte-target drugs, plant-based bioactive compounds and techniques for treating atherosclerosis and associated disorders, further research is required to explore their therapeutic activities at cellular and molecular levels.

## 6. Conclusions

In this review, we described the involvement of pericytes in the pathological processes associated with atherosclerosis development and progression. Multiple risk factors (such as diabetes mellitus, arterial hypertension, shear stress and hypercholesterolaemia) may stimulate atherosclerosis development through endothelial damage, pericyte dysfunction and decrease vascular stability. Advanced stages of atherogenesis strongly correlate with decreased pericyte coverage and atherosclerotic plaque progression, instability, and rupture. It total, we could specify several crucial functions of PCs in AS development and progression:

- PCs facilitate proper neovessel development and maturation
- PCs prevent matrix degradation and maintain vascular stability
- PC–PC and PC–EC communication, PC recruitment and stabilisation are important for neovessels’ permeability, fragility, and impaired perfusion
- PCs regulate mineralisation and osteogenic differentiation of human mesenchymal stem cells and of SMC

Recent studies show that several well-known and effective AS treatments provide pericyte-mediated anti-atherosclerotic, anti-inflammatory and anti-apoptotic effects. In this respect, pericytes represent an obvious target to prevent atherosclerotic plaque progression, decrease plaque neovessels’ leakiness, and increase plaque stability. However, before human clinical trials and commercially available treatments can be developed and fully exploited, further research is required to better understand pericytes’ biology in normal and pathological conductions.

## Data Availability

Not applicable.

## Abbreviations

ACS	acute coronary syndrome
ADAMTS	disintegrin-like and metalloproteinase with thrombospondin motif
AGEs	advanced glycation end products
Ang-1	Angiopoietin 1
AS	atherosclerosis
BAX	Bcl-2-associated X protein
CD13	cluster of differentiation 13
CVCs	calcifying vascular cells
DLK-1	delta-like homolog 1 or Endosialin
Dll4	Delta Like Canonical Notch Ligand 4
DM	diabetes mellitus
DN	diabetic nephropathy
DR	diabetic retinopathy
EC	endothelial cells
ECM	extracellular matrix
eNOS	Endothelia Nitric Oxide Synthase
FGF	fibroblast growth factor
HbA1c	glycosylated haemoglobin
HIF-1	Hypoxia inducible factor-1
HPSCs	human pluripotent stem cells
IPH	intraplaque haemorrhage
KLF2	Kruppel like Factor 2
MMP	matrix metalloproteinases
MSCs	multipotent mesenchymal stem cells
NF-κB	nuclear factor-kB
NG-2	Neuron-glial antigen 2
Ninj1	Ninjurin 1 or Nerve injury-induced protein 1
Notch1	Notch homolog 1
OM	osteoid metaplasia, or bone-like vascular calcification
OPG	osteoprotegerin
PDGF	platelet-derived growth factor
PDGFR-β	platelet-derived growth factor receptor
RAAS	Renin-Angiotensin-Aldosterone system
RBC	Red blood cells
RGS 5	regulator of G protein signalling 5
SMCs	smooth muscle cells
TAZ	transcriptional coactivator with PDZ-binding motif
TGF-β	transforming growth factor β
Tie 2	Tyrosine-Protein Kinase Receptor TIE-2
TIMP	tissue inhibitors of matrix metalloproteinase
TNF-α	tumor necrosis factor alpha
VC	Vascular calcification
VEGF	vascular endothelial growth factor
VV	vasa vasorum
YAP	Yes-associated protein
α-SMA	smooth muscle α-actin
